# Structural effects and lymphocyte activation properties of self-assembled polysaccharide nanogels for effective antigen delivery

**DOI:** 10.1038/s41598-018-34885-8

**Published:** 2018-11-07

**Authors:** Risako Miura, Yoshiro Tahara, Shin-ichi Sawada, Yoshihiro Sasaki, Kazunari Akiyoshi

**Affiliations:** 1 Department of Polymer Chemistry, Graduate School of Engineering, Kyoto University, Katsura, Nishikyo-ku, Kyoto, 615-8510 Japan; 2 Present Address: Department of Applied Chemistry, Graduate School of Engineering, Kyushu University, Motooka 744, Nishi-ku, Fukuoka, 819-0395 Japan

## Abstract

The success of immunotherapeutic vaccines is often limited by their inability to activate the cytotoxic T lymphocyte (CTL)-inducing Th1 pathway. We investigated the ability of self-assembled nanogels (CHP or CH-CDex) to activate this pathway, and characterised them chemically and biologically. Once loaded with antigen (ovalbumin, OVA) their OVA encapsulation and dissociation rates suggested the possibility of effective antigen delivery. The DC2.4 dendritic cell line took up either vaccine time-dependently, but both vaccines required CpG DNA for class I MHC presentation. The nanogel vaccines interacted with RAW264.7, a Balb/c mouse-derived macrophage cell line, and co-localised with lysosomes, suggesting their endocytotic internalization in RAW264.7. Both vaccines activated CTLs better than OVA alone. Unlike OVA alone, the nanogel vaccines induced IgG2a antibody production in mice, whereas the former induced IgG1 antibodies. OVA-nanogel delivery to the draining lymph nodes (DLNs) was higher than that for OVA alone, reaching a deeper medullary area. Furthermore, Langerin^+^ CD103^+^ DCs interacted with the nanogel vaccines effectively, which is a subset of cross-presentation DC, in the DLNs. The nanogel vaccines each had good anti-tumour efficacy in OVA tumour-bearing mice compared with the OVA alone. Thus, CHP and CH-CDex nanogels should be investigated further because of the great potential they offer for immunotherapy.

## Introduction

Immunotherapy, one of the more recently developed and promising methods for cancer treatment, has fewer side effects than chemotherapy using anticancer drugs^1^. Some prophylactic cancer vaccines that target the viral origin of cancers, including human papillomavirus, have been developed and are succeeding in preventing the cancers associated with viruses by inducing antigen-specific antibodies. However, cancer vaccine treatments are not always successful because most of them only activate the Th2 pathway, and activating the Th1 pathway to induce cytotoxic T lymphocyte (CTL) remains difficult. In most cases, after vaccination, exogenous antigens are internalized to antigen presenting cells (APCs) by endocytosis and are presented by the major histocompatibility complex (MHC) class II pathway, which triggers helper T cell-mediated humoral immunity and antibody induction. To present antigens to the MHC class I pathway, which activates cytotoxic T lymphocyte (CTL)-mediated cellular immunity, exogenous antigens should be delivered to the cytosol^2^. Recently, the delivery of antigens to draining lymph nodes (DLNs) has gained growing interest in immunology^3,4^ because of the many types of immune cells they contain and because T cell and B cell activation occurs within them. Some APCs show high cross-presentation, thereby enabling antigen presentation to the MHC class I pathway^5,6^. Therefore, targeting the delivery of antigens to DLNs and controlling antigen uptake to specific APCs within them should be an effective approach for vaccinology. To improve CTL activation via DLN targeting, various antigen delivery carriers such as liposomes^7^, polymer nanoparticles^8^, polymer microparticles^9,10^, polymersomes^11,12^ and hydrogels^13^ have been developed^14^. The efficiency of transfer from the injection site to the lymph nodes depends on the size and rigidity of the nanoparticles^15–17^. Smaller nanoparticles (<100 nm) are passively internalized in the lymph vessels and are effectively moved to the DLNs after subcutaneous injection with them. Smaller nanoparticles can be retained for long periods in the lymph nodes through their interactions with resident APCs. In contrast, larger particles are less efficient at reaching the lymph nodes because the extracellular matrix hinders their internalization, meaning that they need to interact with resident APCs at the injection site to internalize within the lymph vessels. In terms of cellular uptake, APCs take up small or rigid nanoparticles effectively, because of the size of the coated vesicles for endocytosis or activation of the actin filament assembly. Thus, nanoparticle structure is an important factor for controlling immune-system activation.

**Figure 1:**
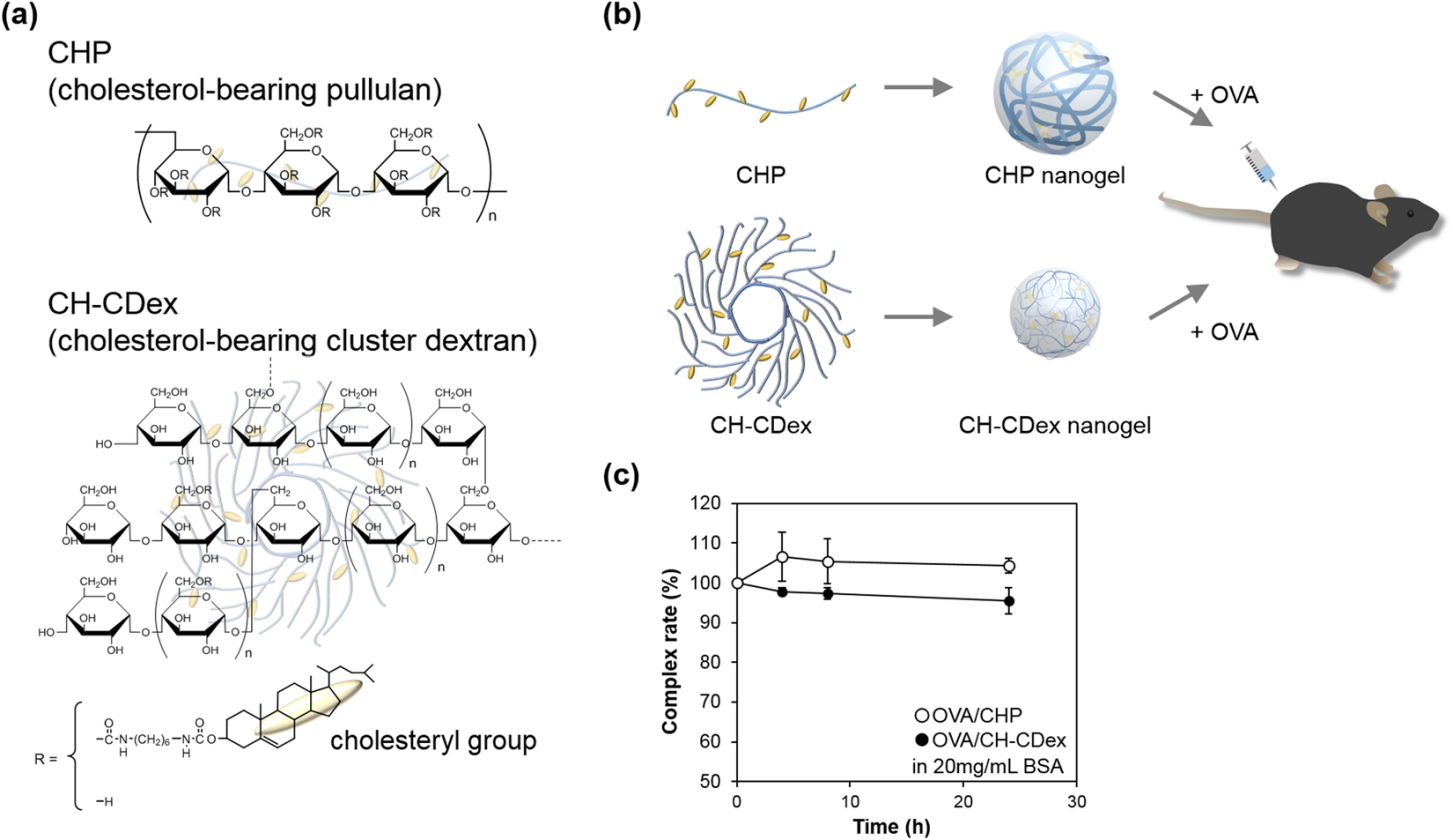
Nanogel vaccine preparation. (**a**) Chemical structures of CHP and CH-CDex. (**b**) A schematic overview of this study. (**c**) Release of OVA from CHP nanogel (open) or CH-CDex nanogel (closed) in the presence of 20 mg/mL of BSA.

We have previously reported that nanogels can act as effective antigen carriers^18^. Cholesterol-bearing polysaccharides (e.g., pullulan^19^, cluster dextrin^20^ and mannan^21^) form nano-sized gels (nanogels) in water via their hydrophobic interactions with cholesterol^22^. The self-assembled nanogels trap proteins via the hydrophobic interactions occurring between the hydrophobic domains in proteins and the physical cross-linking points in the nanogels, and they are released by exchanging their cargo with other proteins (via a chaperon-like activity). Notably, CHP nanogels can activate the humoral and cellular immune systems alike^18,23–25^. CHP nanogels do not interact with non-immune cells, but their high colloidal stabilities and hydrophilic surfaces confer an immune stealth-like ability on them. This ability enables CHP nanogels to deliver antigens to lymph nodes and activate MHC class I presentation via cross-presentation on APCs^26^. Here, we focused our attention on the structural effects of polysaccharides in self-assembled nanogels and lymphocyte activation. The cholesterol-bearing cluster dextrin (CH-CDex) nanogel, a highly branched polysaccharide, was selected because it is a relatively small, rigid nanoparticle in comparison with the CHP nanogel (Fig. 1a). CH-CDex contains about 2.5 times the number of cross-linking points, and a sugar density about 3 times higher than CHP^20^. Both nanogels are approximately 30 nm in diameter without cargo, which are suitable sizes for transfer to the lymph nodes. We evaluated the ability of CHP and CH-CDex nanogels as antigen carriers for vaccination, including CTL activation, antibody production and anti-tumour efficacy. Our discussion of the results is focused on the delivery of antigens to lymph node immune cells.

## Results

### Preparing the nanogel vaccines for use

CHP contains 1.2 cholesteryl groups and CH-CDex contains 3.8 cholesteryl groups per 100 glucose units. CH-CDex has a branched structure and high solubility, which enables more cholesteryl groups to be substituted into the polysaccharide^20^. CHP and CH-CDex both form nanogels via hydrophobic interactions with cholesteryl groups in water.

The diameters of CHP and CH-CDex nanogels in phosphate-buffered saline (PBS) were 32 nm and 23 nm, respectively, after sonication, as measured by dynamic light scattering (DLS) (Table 1). After complex formation with ovalbumin (OVA), their diameters became 63 nm (OVA/CHP) and 22 nm (OVA/CH-CDex), respectively, and both nanogels had non-ionic surfaces. The CH-CDex nanogel had more hydrophobic groups within it compared with the CHP nanogel, and the cholesteryl groups enabled it to form smaller-packaged polysaccharides. By forming a complex with OVA, the CHP nanogel became larger. In contrast, the CH-CDex nanogel-OVA complex formation occurred without any increase in diameter. This results from the CH-CDex nanogels having larger hydrophobic domains with physical cross-linking points inside them, so that each nanogel possibly retains its diameter even after encapsulating proteins via hydrophobic interactions. The percentages of the OVA–nanogel complexes were determined by size exclusion chromatography (SEC).

**Table 1:** Nanogel characterization (n = 3).

	Diameter (nm)	With OVA		
		Diameter (nm)	Zeta potential (mV)	Complexation rate (%)
CHP	32.1 ± 5.5	63.0 ± 1.3	−1.8 ± 0.5	80.9 ± 1.4
CH-CDex	23.4 ± 5.3	22.7 ± 0.8	−2.6 ± 0.8	67.0 ± 2.2

The OVA alone and nanogel-complex retention times were 10 min and 6–8 min, respectively (Supporting information: Fig. S1). Comparing these peak areas, the percentages were determined as 81% (OVA/CHP) or 67% (OVA/CH-CDex). Although OVA/CH-CDex was less than half the diameter of OVA/CHP after complexation with OVA, the CH-CDex nanogel displayed only a 14% lower protein encapsulation capacity. The branching structure of cluster dextrin and higher substitution rate for the cholesterol group, which works as a physical crosslinking point, can affect the smaller packing capacity of the CH-CDex nanogel, even with proteins.

### OVA release from the nanogel complexes

As we have reported previously, self-assembled nanogels can capture proteins like insulin and bovine serum albumin (BSA) stably in their hydrogel networks. However, in the presence of other proteins, the captured protein was released from the nanogel by a protein exchange reaction^27^. Differences in the release rates were attributed to the interactions between the cholesteryl groups inside the nanogels and the hydrophobic domains of the captured proteins. Therefore, to investigate whether differences occur in the OVA release behaviour between CHP and CH-CDex nanogels, OVA/CHP and OVA/CH-CDex complexes were mixed with 20 mg/mL of BSA at 37 °C (Fig. 1c). The result differed to that of our previous study where insulin and BSA were used, and the protein exchange reaction was not observed in either of the nanogel complexes for 1 day. In the cases of insulin and BSA, both are relatively hydrophobic proteins, a property that enables them to be encapsulated by straightforward mixing of the native protein and the nanogel. Conversely, as a hydrophilic glycoprotein, OVA protein requires urea-mediated denatured procedures to form complexes with nanogels. The hydrophobic interaction between denatured OVA and nanogels was stronger than the conventional nanogel complexes seen with insulin and BSA. This result suggests that OVA will not be released from nanogels while they move through the lymph vessels to the lymph nodes.

### Interactions of the nanogel vaccines with APCs

**Figure 2:**
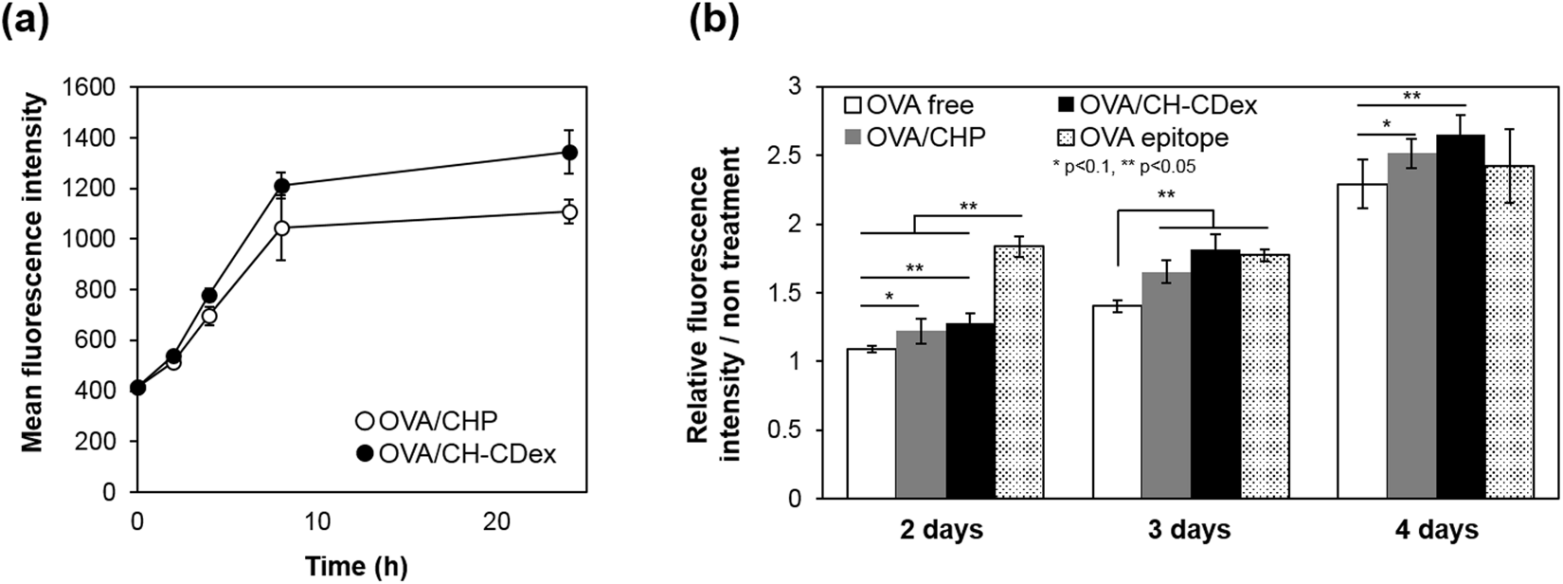
Interaction of nanogel vaccines with DC2.4 cells. (**a**) Time-dependent uptake of OVA-Cy5.5/CHP (open) and OVA/Cy5.5/CH-CDex (closed) by DC2.4 cells. (**b**) Time-dependent relative presentation of the OVA epitope (SIINFEKL) on MHC class I molecules, as evaluated by OVA alone (white), OVA/CHP (gray), OVA/CH-CDex (black) or OVA epitope (SIINFEKL) (dot) using flow cytometry.

We next evaluated how the nanogel vaccines interacted with DC2.4 cells, a dendritic cell line derived from C57BL mice. OVA-Cy5.5/CHP or OVA-Cy5.5/CH-CDex was added to the DC2.4 cells for 24 h in RPMI medium containing 10% foetal bovine serum (FBS), and the fluorescence intensity of the cells was measured by flow cytometry (Fig. 2a). The nanogel vaccine uptake by the DC2.4 cells occurred in a time-dependent manner. The uptake became saturated at around 8 h, with no difference in the general profiles of the nanogels. Both nanogels have hydrophilic surfaces, and this caused a gradual interaction with the DC2.4 cells.

The interactions of the vaccines with RAW264.7 cells, a macrophage cell line derived from Balb/c mice, were observed using confocal laser microscopy (Supporting information: Fig. S2). Nanogel vaccines were added to the RAW264.7 cells with CpG ODN for 24 h, and lysosomes were stained with LysoTracker Green. We found that the OVA-specific fluorescence overlapped with the positions of the lysosomes for both of the nanogel vaccines. This result suggests that the nanogel vaccines were internalized into the cells by endocytosis.

The OVA release from the nanogels after the uptake of the DC2.4 cells was evaluated using DQ-OVA-loaded nanogel vaccines (Supporting information: Fig. S3). DQ-OVA shows bright fluorescence only when hydrolysis of OVA occurs. The fluorescence intensity of hydrolysed OVA increased with both nanogel vaccines in time-dependent manner, and it suggested that CH-CDex nanogel released OVA faster than CHP nanogel. This result indicates that the complexed OVA was released from nanogels and the OVA was hydrolysed by protease in the cells.

### *In vitro* antigen presentation assay

In general, endogenous antigens that are presented to MHC class I molecules activate CTLs, whereas exogenous antigens that are delivered via endocytosis to APCs and are presented to MHC class II molecules activate the antibody production pathway. Thus, it is difficult for vaccines to activate CTL. However, some APCs have cross-presentation abilities, meaning they can present endogenous antigens to MHC class I molecules. DC2.4 is a dendritic cell line with a known cross-presentation ability^28^. The MHC class I presentation of the OVA epitope (SIINFEKL) by nanogel vaccines was evaluated using through flow cytometry via a fluorescently-labelled antibody against H-2K^b^ bound to SIINFEKL peptides.

Using the fluorescence intensity of non-treatment cells as the standard, we compared the fluorescence intensity of each sample (Fig. 2b). All samples showed a time-dependent increase in OVA epitope presentation on MHC class I molecules. DC2.4 was activated by the nanogel vaccines more than by OVA alone. Also, DC2.4 was not activated by the nanogel vaccines without CpG (Supporting information: Fig. S4), indicating that the combination of nanogel vaccines and CpG was necessary for MHC class I presentation. From these results, we conclude that our nanogel vaccines have the ability to activate antigen presenting cells and induce the MHC class I presentation pathway.

### CTL activation

**Figure 3:**
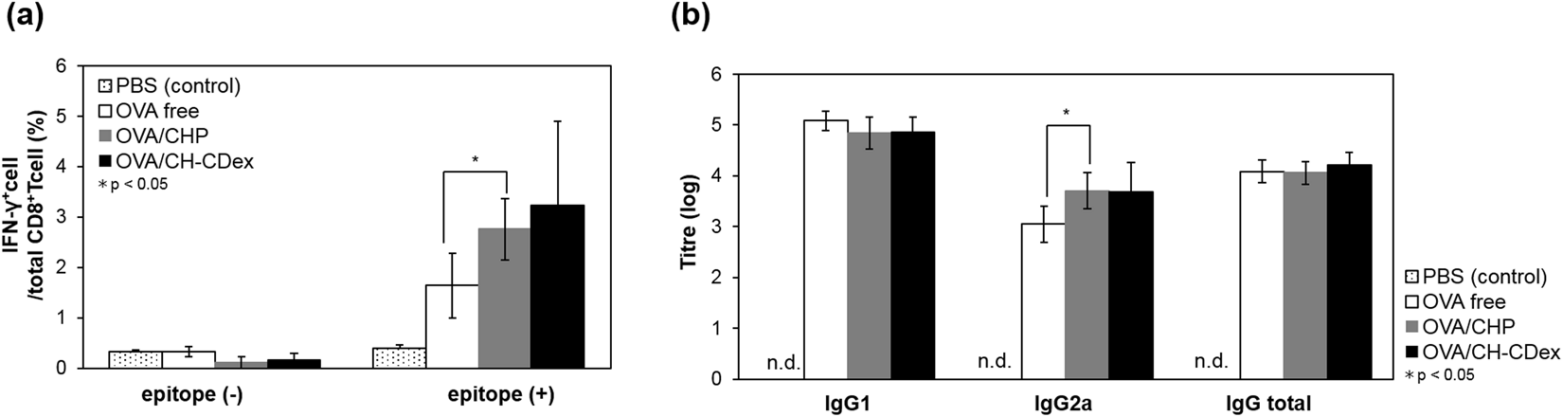
Evaluation of the immune activation abilities of the nanogel vaccines *in vivo*. (**a**) Ratio of OVA activated CTL to total CD8a^+^ cells by PBS (dot), OVA alone (white), OVA/CHP (grey) or OVA/CH-CDex (black) analysed by flow cytometry. (**b**) Total serum titres for IgG1, IgG2a and IgG in C57BL/6J were analysed by ELISA.

To evaluate the vaccination abilities of the OVA-loaded nanogels, we studied adaptive immunity against them in mice. Nanogel vaccines were injected subcutaneously into C57BL/6J mice (twice a week over 2 weeks), after which the spleen cells were collected and the OVA-specific CTL activation rate was evaluated by flow cytometry. Splenocytes were stained with an anti-CD8 antibody, and the OVA-specific activated CTLs were stained with an anti-IFN-γ antibody after H-2K^b^ OVA epitope peptide stimulation. The percentages of the activated CTLs with OVA alone, OVA/CHP or OVA/CH-CDex were 1.6, 2.8, 3.2%, respectively, and both CHP and CH-CDex nanogel vaccines showed effective CTL activation compared with OVA alone (Fig. 3a). Unlike the situation where a CHP nanogel vaccine was shown to effectively activate CTL in mice using tumour-related antigens, and was tested in a clinical trial as a therapeutic cancer vaccine^24^, this is the first report to show that nanogels enhance CTL activation using OVA as the antigen. Additionally, our CH-CDex nanogel vaccine, which showed almost same CTL activation ability as the CHP nanogel, is expected to work as an effective antigen carrier.

### Antibody production

OVA-specific antibody production was evaluated by enzyme-linked immunosorbent assay (ELISA). After immunization with the vaccines, serum was collected from blood samples from the C57BL/6J mice, after which the IgG1, IgG2a and IgG titres of the anti-OVA serum antibodies were evaluated (Fig. 3b). The nanogel vaccines did not differ significantly for each antibody type. This result indicates that the polysaccharide structure *per se* does not affect the antigen presentation pathway for MHC class II or affect the antibody production pathway. By comparing the nanogel vaccines with the OVA alone, the former were able to effectively induce IgG2a antibody production (Fig. 3b). Contrastingly, the OVA alone induced IgG1 antibody production. IgG1 antibodies are produced by Th2 cell activation, whereas IgG2a is activated by Th1 cells. Furthermore, the IgG2a antibody production pathway is associated with CTL activation whereby cytokines are released to activate CD8^+^ T cells. CTL activation and antibody production studies have shown that nanogel vaccines release the antigen so that it is presented to both MHC class I and II, thereby enhancing the effectiveness of the Th1-type IgG2a antibody production pathway related to CTL activation. Other self-assembled nanogels lacking a CpG DNA adjuvant were reported to elicit IgG2a production; thus it is suggested that the CpG DNA adjuvant has an effect on humoral immune system activation, and nanogels enhanced the efficacy^29^. In this study, the nanogels do not interact with CpG-DNA because the nanogels are non-ionic. They were delivered to lymph nodes separately by subcutaneously injection. In our previous work^26^, we have shown that CpG-DNA accumulated to lymph nodes and interacted with dendritic cells or macrophages via subcutaneously injection.

### Lymph node observations

**Figure 4:**
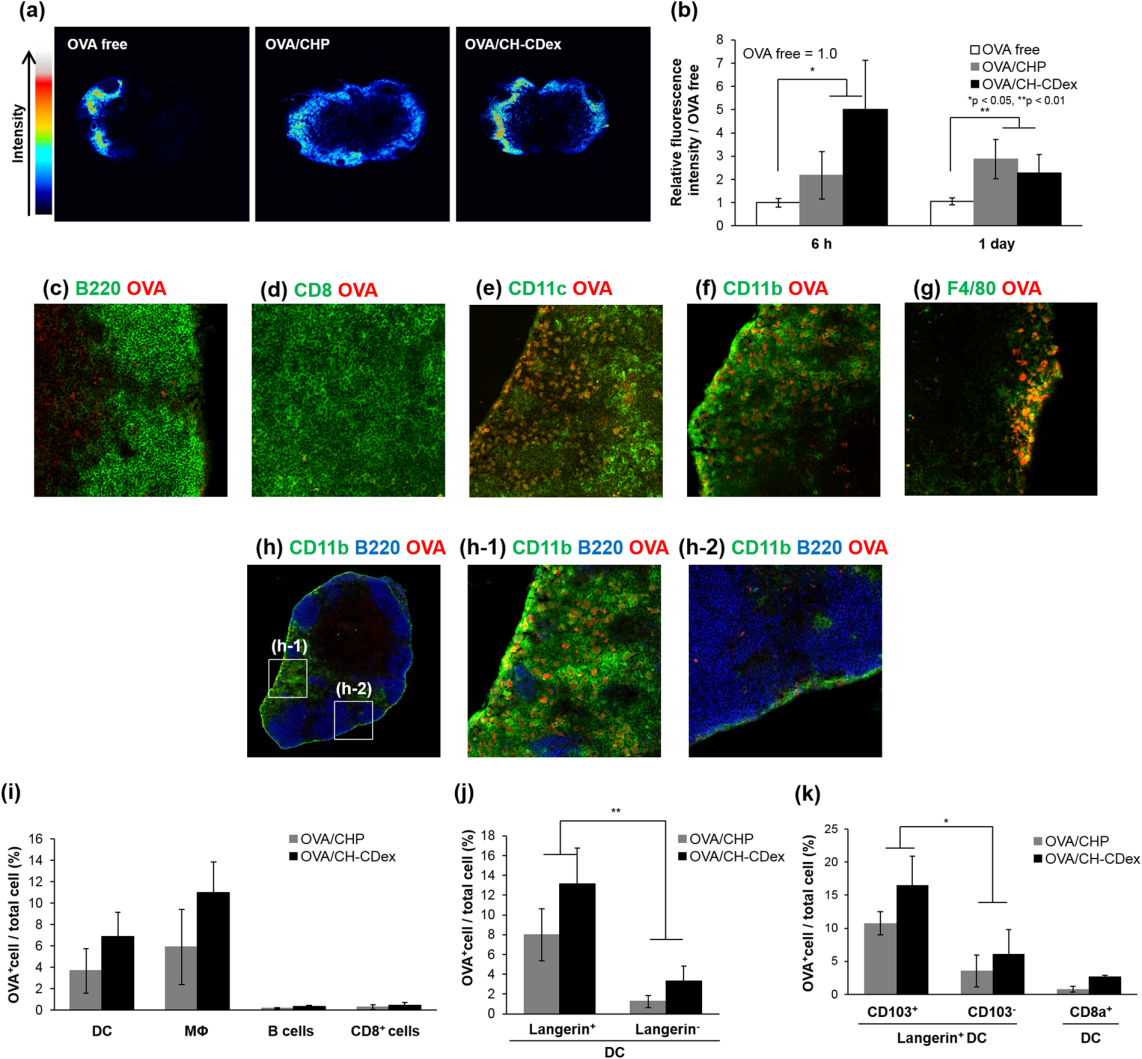
Effective antigen delivery to DLNs by nanogel vaccines. (**a**) Distribution of OVA-Cy5.5, either free or in nanogel complexes in lymph node tissues 6 h after administration, as observed by CLSM. (**b**) Fluorescence intensity of OVA-Cy5.5 in lymph node tissue as measured by IVIS. (**c**–**h**) Distribution of immune cells and OVA-Cy5.5/CH-CDex in lymph nodes 6 h after administration, as observed by CLSM. (**c**) B220, (**d**) CD8, (**e**) CD11c, (**f**) CD11b and (**g**) F4/80 are all shown in green whereas OVA is shown in red. (**h**) The whole image of a lymph node (B220: blue, CD11b: green, OVA: red) and (**h**-**1**) an enlarged view of the medullary area and (**h**-**2**) subcapsular area. (**i**–**k**) Internalization of OVA-Cy5.5/CHP (grey) or OVA-Cy5.5/CH-CDex (black) to (**i**) antigen presenting cells such as dendritic cells (DCs), macrophages (MΦ) or B cells, and CD8^+^T cells. (**j**) DCs with or without Langerin, (**k**) Langerin^+^ DCs with or without CD103, and CD8^+^ DCs.

Delivering antigen to various immune cells reside within the lymph nodes is important for adaptive immunity to occur. Thus, we observed antigen delivery to the lymph nodes by confocal laser scanning microscopy (CLSM) and the *In Vivo* Imaging System (IVIS). Focusing on the DLNs (Fig. 4a,b), the antigen was delivered more efficiently by using the nanogels as the antigen carriers rather than OVA alone, and the CH-CDex nanogel was the more effective antigen delivery system than CHP nanogel at the 6 h time point. It is reported that the smaller particles accumulate to lymph nodes and more strongly interact with antigen presenting cells via subcutaneously injection^17,30^. The faster accumulation and clearance of CH-CDex/OVA complex nanogels (22 nm) than CHP/OVA complex nanogels (63 nm) were probably attributed to the sizes of nanogels. These results show that CH-CDex nanogels acted as novel antigen carriers and succeeded in delivering more antigen to the lymph nodes than OVA alone.

### Delivery to lymph node immune cells

To evaluate the internalization of antigens to antigen presenting cells including B cells, CD8^+^ cells dendritic cells and macrophages, a section from the lymph nodes was stained with cell-surface markers. Figure 4c–g shows that after OVA antigen delivery by the CH-CDex nanogel, the fluorescence from OVA-Cy5.5 did not co-localize with B cells (B220^+^) or CD8^+^ T cells, but did co-localized with the dendritic cells (CD11c^+^) and macrophages (CD11b^+^ or F4/80). Additionally, Fig. 4h shows that the macrophages capturing OVA-Cy5.5 were mainly distributed in the medullary area compared with the subcapsular area. Medulla macrophages are known to have a high cross-presentation ability^26^, and in the present study they were able to deliver antigens into a deeper area of the lymph nodes via the CH-CDex nanogel, and this was to be led to effective CTL activation.

Flow cytometric analysis was used to determine the uptake of OVA-Cy5.5 by dendritic cells (DC, CD11c^+^), macrophages (CD11b^+^F4/80^+^), B cells (B220^+^) or CD8^+^T cells (Fig. 4i). Both nanogel vaccines interacted with dendritic cells and macrophages, but not with B cells or CD8^+^T cells. As reported before, CHP nanogels exhibit immunological stealth and prevent nonspecific binding to the extracellular matrix and cells^26^. This result shows that CH-CDex can also deliver antigens to medullary macrophages. Our analysis also focused on the interactions between the OVA-loaded nanogels with Langerin^+^ DCs. As Fig. 4j shows, the OVA antigen was internalized by the Langerin^+^ cells via nanogel vaccine delivery to a greater extent than the Langerin-negative cells. Langerin^+^ DCs are a type of migratory cell that move from the dermis to the lymph nodes, and one subset (CD103^+^) of Langerin^+^ DCs show cross-presentation ability^5,6^. As shown in Fig. 4k, the nanogel vaccines were able to interact effectively with Langerin^+^ CD103^+^ DCs. However, the delivery of OVA-Cy5.5 to DCs expressing surface CD8, which have cross-presentation ability, was low. These results suggest that Langerin^+^ CD103^+^ DCs were to be able to affect CTL activation via cross-presentation.

### Immunotherapy in tumour-bearing mice

**Figure 5:**
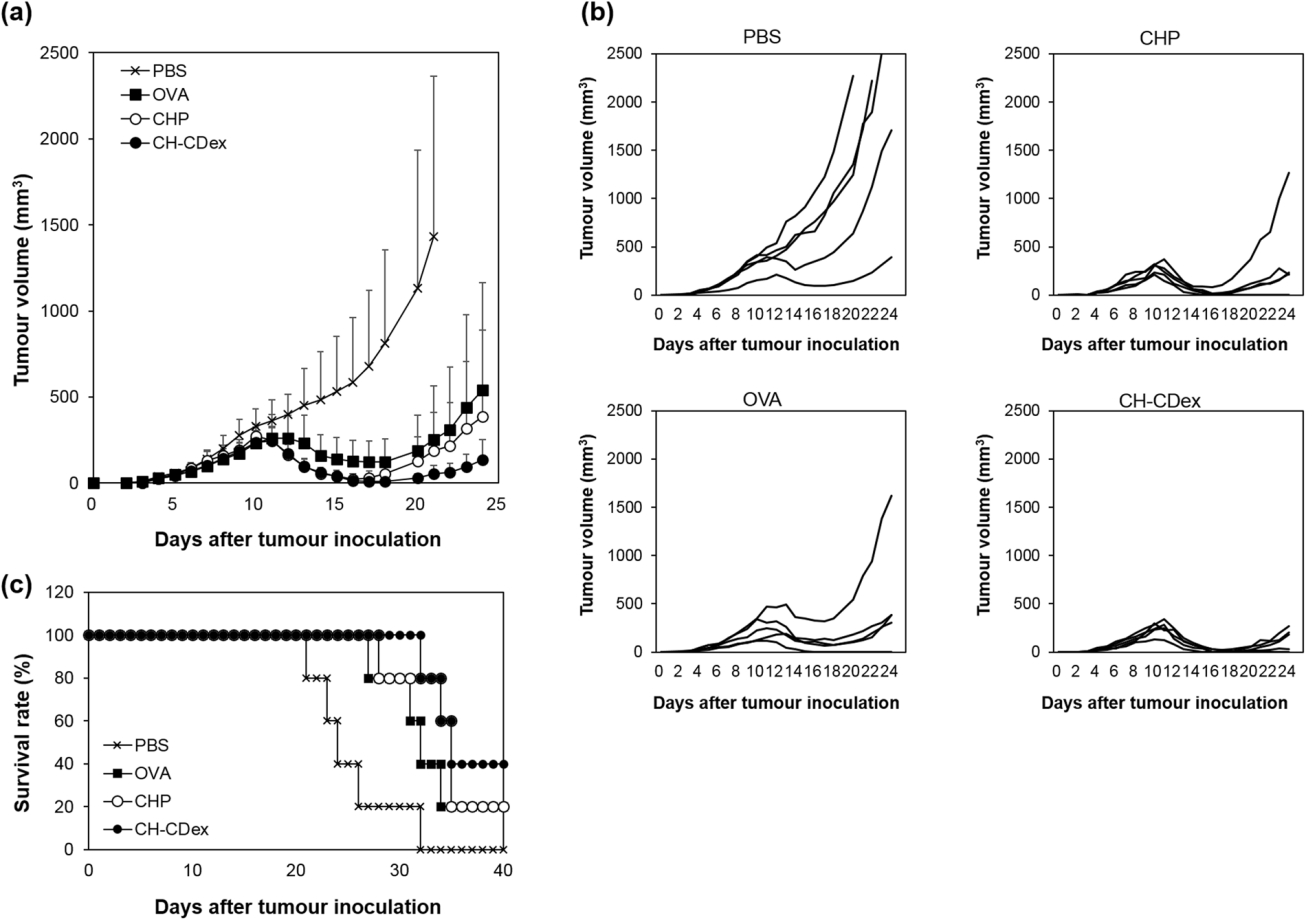
Anti-tumour efficacy of nanogel vaccines. (**a**) Tumour volume of C57BL/6J mice inoculated E.G7-OVA cells, vaccinated PBS as control (cross), OVA (square), OVA/CHP (open circle) or OVA/CH-CDex (closed circle) on 5 and 9 days after tumour inoculation. (**b**) Tumour volume of each mice and (**c**) survival rate of each group.

To evaluate the anti-tumour efficacy of the nanogel vaccines, the vaccines were injected subcutaneously into tumour-bearing mice. E.G7-OVA cells, which are a derivative of EL4 cells (a C57BL/6 mouse lymphoma cell line) and secrete OVA constitutively, were inoculated into the C57BL/6J mice, and OVA alone, OVA/CHP or OVA/CH-CDex with CpG was administered by injection on days 5 and 9 after tumour inoculation. As a control, PBS was administered by injection to monitor the natural growth of the E.G7-OVA cells. Figure 5a shows the average tumour volume for each group, and Fig. 5b shows the tumour volumes in each mouse. After the first injection, all the vaccinated groups started to suppress the growth of their tumours. After the second injection, the immune system was boosted and the tumour volume decreased more sharply in the mice that received the nanogel vaccines than those that received OVA alone. At around 16–18 days after tumour inoculation, while OVA alone had failed to decrease the tumour volume completely, the OVA/CHP and OVA/CH-CDex vaccines succeeded in decreasing the tumour size and some of the tumour-bearing mice were cured at this moment in time. In the PBS injection group, some mice showed tumour-growth suppression at around 12 days post injection. This suggests that some mice were able to adopt anti-tumour immune activation naturally, but it was not enough to affect a cure. The tumour sizes after 24 days post-injection (Fig. 5a,b), and the survival rates of the mice (Fig. 5c), showed that the CH-CDex nanogel had a somewhat better effect against tumours that those seen in the other mouse groups. This finding is most likely related to CTL activation (Fig. 3a), in which the CH-CDex nanogel showed the highest potential to produce antigen specific CTLs.

## Discussion

CHP and CH-CDex nanogel vaccines were prepared and their sizes were readily controlled by changing the structure of polysaccharide, such as linier or branched, and number of substituted cholesteryl units. The results of our *in vitro* studies showed that antigen presentation to the MHC class I pathway via the nanogel vaccines is higher than that for OVA alone. Furthermore, the *in vivo* vaccination study showed that the CHP and CH-CDex nanogel vaccines succeeded in inducing OVA-specific CTLs and antibodies. Notably, the nanogel vaccines were able to activate OVA-specific CTLs more effectively than OVA alone. The *in vivo* study of antigen delivery to the lymph nodes showed that the delivery of OVA to the DLNs via the nanogel vaccines was higher than that for OVA alone. The immune activation effect did not depend on the size of the nanogels, but the CH-CDex nanogel succeeded in delivering OVA to the lymph nodes in the shortest time, and also facilitated OVA infiltration into the deeper medullary area. In the lymph nodes, the nanogel vaccines internalized in the medullary macrophages and DCs, especially the migratory DCs. Langerin^+^ CD103^+^ DCs are an important DC subset, which show cross-presentation and were able to capture the antigens delivered by nanogels. The above-mentioned qualities of the nanogel vaccines acted to enhance the efficacy of the anti-tumour therapy, allowing them to succeed in treating the E.G7-OVA tumours in the experimental mice. These results suggest that self-assembly nanogel-based antigen delivery is a promising vaccination platform that merits further investigation in the context of therapeutic and prophylactic vaccine development.

## Methods

### Supplementary material

The origins and manufacturers of the materials not mentioned below can be found in the online supplementary materials section.

### Ethics statement

All experiments were performed in accordance with the relevant guidelines and regulations. All animal experiments were approved by the Ethics Committee for Animal Welfare of Kyoto University, Japan.

### Cell cultures and animals

DC2.4 cells were maintained in RPMI 1640 medium (Gibco, Carlsbad, CA, USA) containing 10% FBS and 1% penicillin-streptmycin. The cells were kindly supplied by Professor Shiku (Mie University, Japan). Female C57BL/6J mice were purchased from SLC Co. Japan (Shizuoka, Japan).

### Nanogel vaccine preparation and OVA release

Nanogel vaccines were prepared as follows^26^: CHP (*M*_w_ = 100,000, 1.2 cholesteryl groups per 100 glucose units) and CH-CDex (*M*_w_ = 100,000, 3.8 cholesteryl groups per 100 glucose units) polymers were individually dissolved in PBS containing 6 M urea by stirring at room temperature overnight. OVA was dissolved in PBS and mixed with each nanogel in 6 M urea at 37 °C for 24 h, followed by dialysis against PBS to remove the urea. The resulting OVA/CHP and OVA/CH-CDex solutions were individually filtered through a 0.22 μm membrane and then stored at 4 °C until use. The final concentrations of the CHP and CH-CDex nanogels were 5 mg/mL each, whereas that of OVA was approximately 0.2 mg/mL. To determine the size of the nanogels and OVA/nanogel complexes, DLS (Zetasizer Nano-ZS; Malvern, UK) measurements were performed. Complexation of the nanogels and Cy5.5-labeled OVA (OVA-Cy5.5) was confirmed using SEC with a TSK-gel G3000SWXL column (TOSOH Co., Tokyo, Japan). Each nanogel vaccine was mixed with 20 mg/mL BSA at 37 °C for 1 day. The OVA release rate from the nanogels was confirmed by SEC.

### Interaction of nanogel vaccines with APCs

DC2.4 cells (2 × 10^5^ cells) were pre-cultured in 12-well plates, and OVA-Cy5.5-loaded nanogel vaccines (2 µg of OVA per well) were added to the medium for up to 24 h, followed by collagenase dispersion. The fluorescence of OVA-Cy5.5 was then measured by flow cytometry (LSR Fortessa cell analyzer, BD Biosciences, San Jose, CA, USA) and the resulting data were analysed using FlowJo 7.6.5 software (Tree Star). For observation, RAW264.7 cells (1 × 10^4^ cells) were pre-cultured in glass-bottomed multi-well plates, and the OVA-Cy5.5-loaded nanogel vaccines (2 µg of OVA per well) were added to the medium for 24 h, followed by lysosome staining with LysoTracker™ Green DND-26 (Thermo Ficher Scientific, Waltham, MA, USA). Next, the fluorescence patterns from OVA-Cy5.5 and the lysosomes were observed by CLSM (LSM780; Carl Zeiss, Oberkochen, Germany). For evaluation of OVA release within cells, nanogel vaccines were prepared using DQ-OVA, which shows bright fluorescence when it hydrolyzed. DC2.4 cells (1 × 10^5^ cells) were pre-cultured in 12-well plates, and DQ-OVA-loaded nanogel vaccines (1 µg of OVA per well) were added to the medium up to 24 h, followed by collagenase dispersion. The fluorescence of DQ-OVA was then measured by flow cytometry (Cytomics FC500, Beckman Coulter, Ink., Brea, CA, USA).

### *In vitro* antigen presentation assays

DC2.4 cells (2 × 10^4^ cells) were pre-cultured in 12-well plates, and OVA alone, nanogel vaccines (2 µg of OVA) or 47 ng of OVA epitope (SIINFEKL) as positive control with 1.25 µg of CpG were added to the medium. The cells were co-cultured for up to 4 days, followed by collagenase dispersion. The OVA epitope presented on MHC classI was immuno-stained with PE (R-phycoerythrin) anti-mouse H-2K^b^ bound to the SIINFEKL antibody. The fluorescence intensities were measured by flow cytometry (Cytomics FC500, Beckman Coulter, Ink., Brea, CA, USA) and the data were analysed using FlowJo 7.6.5 software (Tree Star).

### Mouse immunizations

OVA alone, OVA/CHP or OVA/CH-CDex nanogels were mixed with CpG DNA (0.05 mg/mL) as the adjuvant, and 50 µL was administered subcutaneously to both the right and left side of the tail base of each C57BL/6J mouse. Administration was performed twice a week for 2 weeks, and the results were assessed by CTL and antibody production assays, as described below.

### CTL assays

CTL activation by the nanogel vaccines was evaluated as follows^26^. The spleen was harvested from an immunized mouse and the cell suspension was re-suspended in RPMI 1640. After ammonium-chloride-potassium buffer treatment to lyse the red blood cells, the remaining cells were filtered through a 40-µm cell strainer (BD Biosciences). The cells were incubated with the H-2K^b^ OVA epitope peptide at 37 °C for 1 h, and then with Goldi Plug (BD Biosciences) overnight. The collected cells were stained with fluorescent dye-labelled anti-CD8a or IFN-γ antibodies at 4 °C for 20 min, and then fixed using the Cytofix/Cytoperm kit (BD Biosciences). The fluorescence intensities were measured by LSR (Fortessa cell analyzer, BD Biosciences) and the data obtained were analysed using FlowJo 7.6.5 software (Tree Star).

### Antibody production assays

Blood was collected from the mice and serum was obtained from it by centrifugation. Total IgG, IgG1 and IgG2a serum antibody concentrations were measured by ELISA. Briefly, 5 mg/mL of OVA in PBS was coated onto 96 well-plates and then incubated at 4 °C overnight. After washing with PBST (PBS containing 0.1% Tween 20), 2% BSA in PBS was added as the blocking agent at 37 °C for 2 h. The serum was diluted in 2% BSA, followed by reaction at 37 °C for 2 h. The HRP-labelled anti-IgG1, IgG2a or IgG antibodies were reacted with the antigen at 37 °C for 2 h. TMB Liquid Substrate (Sigma-Aldrich, St. Louis, MO, USA) was added, the plates were incubated at 37 °C for 30 min, and the reaction was stopped by adding 2 M HCl. Antibody–antigen reactions were assessed by measuring their optical density at 450 nm.

### Lymph node observations

OVA-Cy5.5 alone or OVA-Cy5.5-loaded nanogel complexes were administered subcutaneously to mice. After 6 h or 1 day post administration, the fluorescent intensities of OVA-Cy5.5 were observed in the skin or the DLNs of the mice by IVIS (Lumina LT, PerkinElmer, Waltham, MA). After 6 h post administration, the DLNs were collected in normal saline (0.09% saline) and the OVA-Cy5.5 distribution was observed using CLSM.

### Antigen delivery to lymph node immune cells

For immunohistochemistry analysis, frozen sections of the DLNs were prepared 6 h after administration of OVA-Cy5.5 alone or OVA-Cy5.5-loaded nanogel complexes. The sections were fixed with cold acetone, followed by immune staining with fluorescent dye-labelled anti-CD11c, CD11b, CD8 and B220 antibodies. Using CLSM, the distributions of the immune cells and OVA-Cy5.5 were observed. For flow cytometry analysis, the cell suspension was prepared from the DLNs in cold 0.5% BSA 6 h after administration of OVA-Cy5.5 alone or nanogel complexes. The cells were stained with fluorescent dye-labelled anti-CD207, CD11c, CD11b and F4/80, B220, CD103 or CD8a antibodies at 4 °C for 20 min. The fluorescence intensities were measured by flow cytometric analysis.

### Immunotherapy in the tumour-bearing mice

E.G7-OVA cells (1 × 10^6^ cells) were inoculated subcutaneously into the left-hand side of the backs of the C57BL/6J mice. On days 5 and 9 after inoculation, 100 µL of OVA alone (0.2 mg/mL) or nanogel vaccines (5 mg/mL of nanogel and 0.2 mg/mL of OVA) with CpG adjuvant (0.05 mg/mL) were injected subcutaneously into the right-hand side of the mouse backs. As a control, some mice were not immunized so that the natural growth of E.G7-OVA could be monitored. Tumour sizes were monitored from the day of inoculation. Tumour volumes were calculated using the following formula: tumour volume = (major axis) × (minor axis)^2^ × 0.5. Mice were killed for ethical reasons when the tumour volumes became larger than 2500 mm^3^. Each group contained 5 mice.

## Electronic supplementary material

Supplementary information

## Data Availability

All relevant data are available from the authors.
