# Supplementary material for: Structural effects and lymphocyte activation properties of self-assembled polysaccharide nanogels for effective antigen delivery
Source: Sci Rep. 2018 Nov 7;8:16464. doi: 10.1038/s41598-018-34885-8 (PMC6220277; doi:10.1038/s41598-018-34885-8)
Supplement: Supplementary file 1 — Supplementary information [file 41598_2018_34885_MOESM1_ESM.docx]

**Structural effects and lymphocyte activation properties of self-assembled polysaccharide nanogels for effective antigen delivery**

**Authors**

Risako Miura, Yoshiro Tahara^+^, Shin-ichi Sawada, Yoshihiro Sasaki, Kazunari Akiyoshi

Department of Polymer Chemistry, Graduate School of Engineering, Kyoto University, Katsura, Nishikyo-ku, Kyoto 615-8510, Japan.

^+^Current address: Department of Applied Chemistry, Graduate School of Engineering, Kyushu University, Motooka 744, Nishi-ku, Fukuoka 819-0395, Japan.

**Supplementary Information**

**Materials**

CHP and CH-CDex were synthesized as described previously^19,20^. Phosphate-buffered saline, RPMI 1640, fetal bovine serum and penicillin–streptomycin were purchased from Gibco. EndoGrade OVA was purchased from Hyglos GmbH (Bernried, Germany). Urea was purchased from Wako (Osaka, Japan). LysoTracker™ Green DND-26 and Slide-A-Lyzer^™^ Dialysis Cassettes (10K MWCO, 3mL) were purchased from Thermo Fisher Scientific. DQ Ovalbumin (DQ-OVA) was purchased from Invitrogen (Carlsbad, CA, USA). CpG with phosphorothioate modification was purchased by FASMAC (Kanagawa, Japan). PE anti-mouse H-2K^b^ bound to SIINFEKL antibody (25-D1.16), APC anti-mouse CD8 antibody (53-6.7), PE anti-mouse IFN-γ antibody (XMG1.2), PE anti-mouse CD11c antibody (N418), Pacific Blue anti-mouse CD11b antibody (M1/70), PE/Cy7 anti-mouse F4/80 antibody (BM8), PE/Cy7 anti-mouse B220 antibody (RA3-6B2), PE/Cy7 anti-mouse CD103 antibody (2E7), PE/Cy7 anti-mouse CD8 antibody (53-6.7), and the Alexa 488 anti-mouse B220 antibody (RA3-6B2) were purchased from BioLegend (San Diego, CA, USA). The anti-CD207 (Langerin) monoclonal antibody Alexa Fluor 488 (eBioRMUL.2) was purchased from eBioscience (San Diego, CA, USA). GoldiPlug and Cytofix/Cytoperm Kits were purchased from BD Bioscience. Bovine serum albumin and 3,3',5,5'-tetramethylbenzidine Liquid Substrate were purchased from Sigma-Aldrich. Tissue-Tech O.C.T. Compound was purchased from Sakura Finetek, Japan (Tokyo, Japan).


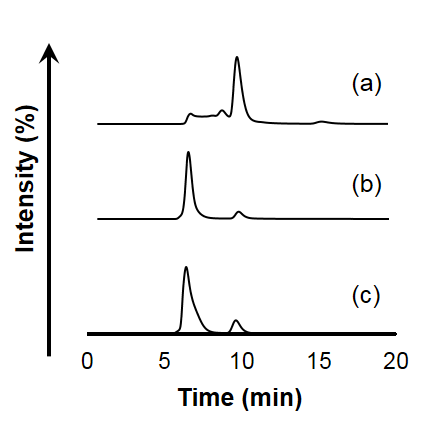


**Figure S1.** Size exclusion chromatogram of **(a)** OVA-Cy5.5, **(b)** OVA-Cy5.5/CHP and **(c)** OVA-Cy5.5/CH-CDex. The complexation rate was determined by using the calibration curve for OVA-Cy5.5.

**
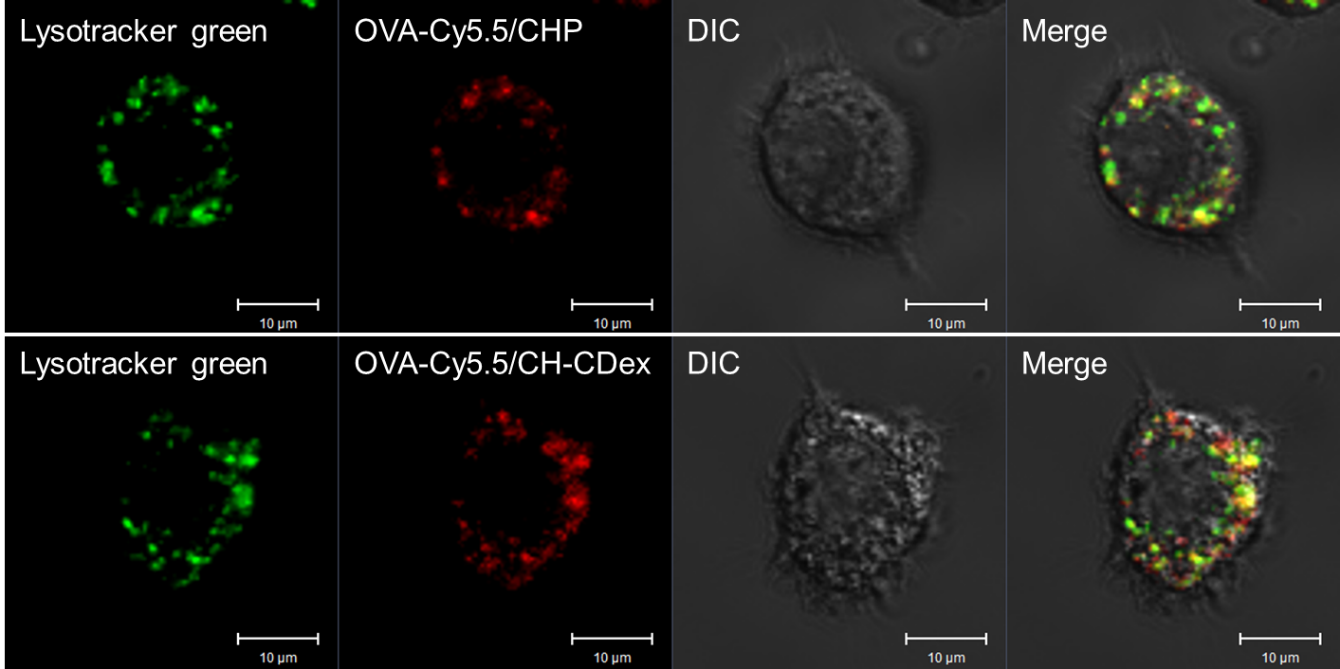
**

**Figure S2.** Vaccine localization in RAW264.7 cells was observed by confocal laser microscopy (upper row: OVA-Cy5.5/CHP, bottom row: OVA-Cy5.5/CH-CDex). DIC, differential interference contrast


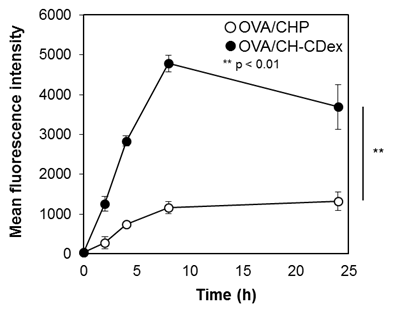


**Figure S3.** Time-course of OVA hydrolysis in DC2.4 cells with OVA/CHP (white) or OVA/CH-CDex (black).

**
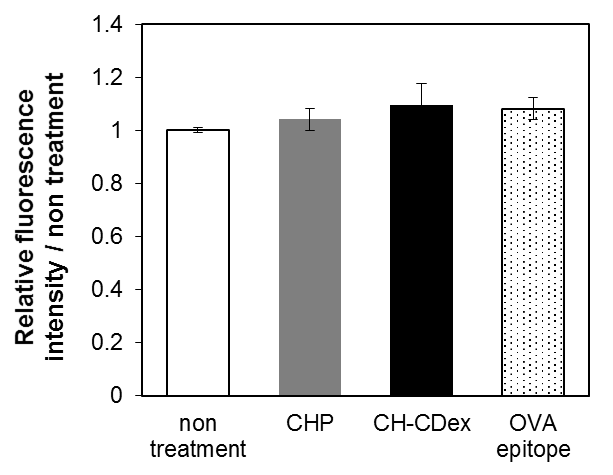
**

**Figure S4.** *In vitro* MHC class I presentation on DC2.4 cells with OVA/CHP (gray), OVA/CH-CDex (black) or OVA epitope (SIINFEKL) (dot) without CpG DNA. DC2.4 cells were co-cultured with the nanogel vaccines for 4 days at 37^o^C.
